# Role of Prophylactic Sartorius Flaps for Preventing Deep Space Infection in Lower Extremity Revascularization

**DOI:** 10.7759/cureus.32228

**Published:** 2022-12-05

**Authors:** Brandon Larson, Joseph DiBello, Logan Erz, David Gothard, Eric Turney

**Affiliations:** 1 General Surgery, Summa Health, Akron, USA; 2 Medicine, Summa Health System, Akron, USA; 3 Medicine, Biostats Inc, East Canton, USA; 4 Vascular Surgery, Summa Health, Akron, USA

**Keywords:** deep space infection, peripheral arterial disease, surgical site infection, prophylactic flap, sartorius flap

## Abstract

Groin infections in vascular surgery are common and compromise the goal of limb preservation. Strategies to prevent deep space infection (DSI) include incision orientation and muscle flaps. Literature evaluating prophylactic flaps preventing DSI is scarce. We aimed to compare prophylactic sartorius flaps to layered closure in preventing readmission for DSI, along with the effect of incision orientation.

This was a retrospective study of vascular surgery patients at a single institution with femoral artery exposure from 2017-2021. Patients with active groin infections were excluded. Prophylactic sartorius flaps were compared to those with layered closure regarding 30-day hospital readmission for DSI. Oblique versus vertical incisions was compared regarding the primary outcome.

Fifty-three patients received sartorius flaps, and 122 received layered closure. Seventy patients had oblique incisions, and 105 patients had vertical incisions. Sartorius flaps had a higher rate of previous groin surgery compared to layered closure (45.3% vs. 24.7%, p<0.01). Vertical incisions had a higher rate of previous groin surgery (38.1% vs. 20.0%, p<0.02), while oblique incisions had a higher rate of obesity (24.3% vs. 8.6%, p<0.01).

There was a lower rate of DSI in sartorius flaps compared to layered closure (1.9% vs. 6.6%, p=2.80), although not statistically significant due to lack of power. There was no difference in DSI in the oblique versus vertical incisions (4.3% and 5.7%, p=0.760).

Patients with prophylactic sartorius flaps experienced fewer DSI, although further evaluation with increased sample size is required for adequate study power. We believe sartorius flaps are a simple solution to prevent groin complications.

## Introduction

Postoperative groin infections in peripheral revascularization surgery occur in up to 30% of patients. These infections have potentially catastrophic postoperative morbidity and threaten the goal of limb preservation. Prior studies have shown groin surgical site infections (SSI) lead to increased hospital length of stay, readmission rates, reoperation, morbidity, and mortality [1]. Groin wound infections range from superficial, involving the skin and subcutaneous tissues, to deep space infections (DSI), involving the tissues immediately surrounding the femoral vessels. DSIs are particularly threatening to a bypass or patch angioplasty and can lead to overwhelming sepsis or anastomotic breakdown. Recognition of the ominous nature of DSIs dates back multiple decades when Samson et al. created a classification system to categorize vascular graft infections based on anatomic depth. In this classification system, Group 1 infection is superficial to the dermis. Group 2 infection is within subcutaneous tissue but does not directly contact the graft. Group 3 infection directly contacts the graft but not the anastomosis. Group 4 infection contacts an anastomosis, but the patient does not have bacteremia or anastomotic bleeding. Group 5 infection surrounds the anastomosis with evidence of sepsis or anastomotic bleeding [2]. 

The Penn Groin Assessment Scale predicts groin complications based on patient risk factors. The scale assigns one point for obesity and two points each for smoking history, reoperation, and the presence of prosthetic graft material for a maximum of seven points. The patient risk is further stratified into low (zero to two), intermediate (three to four), and high (greater than five) risk categories. The scale has shown that patients with higher scores have a higher overall incidence of complications [3]. Additional comorbidities thought to impair patient wound healing and diminish the immune response include chronic kidney disease and immunosuppressive therapy. Reoperative groins involve dissection through existing scar tissue which compromises postoperative wound healing. Obesity and the female gender place a higher distribution of peripheral adipose tissue in the groin region with overlying pannus and increased shearing force on the incision itself [4].

Several strategies have been employed to mitigate these risk factors for wound complications, such as incision orientation. Vertical incisions have been traditionally favored due to ease of vessel access; however, there has been a suggested association with a higher incidence of SSI and seroma formation. This association is attributed to the disparity in skin oxygenation between the medial and lateral sides of the vertical incision. Oblique incisions also run along Langer’s lines, which, in theory, reduces skin tension on the incision [5,6].

More recently, the use of prophylactic local muscle flaps has gained popularity amongst both vascular and plastic surgeons. Muscle flaps are felt to reduce potential space surrounding a graft and provide well-vascularized tissue as a physical barrier to decrease the local bacterial burden. Fischer et al. demonstrated patients with prophylactic flaps had a lower overall rate of wound complications (16.2% vs.. 50.3%; p<0.001) and infections (1.5% vs. 38.5%; p<0.001); however, the authors did not adequately delineate superficial SSIs vs. DSIs. These findings existed despite the flap group having a higher incidence of medical comorbidities compared to the control group [7]. Wallace et al. demonstrated prophylactic muscle flaps were associated with a reduced need for reoperation overall (50.0% vs. 12.2%, p< 0.01) and operative intervention specifically for wound breakdown (71.4% vs. 20.7%, p= 0.02); however, patients with prior groin operations were excluded which is an established risk factor for wound complications [8]. The inclusion of only index revascularization procedures excludes a high-risk patient population, thus diminishing the potential application of prophylactic muscle flaps to the broader vascular surgery patient population. Further, the patients routinely require five days of bedrest as standard postoperative protocol. In our experience, this bedrest period is unnecessary and significantly increases a patient’s hospital length of stay.

We present a novel study that evaluates the use of both local muscle flaps and incision orientation in a diverse vascular patient population, including index procedures and reoperative groins. The primary purpose of this study was to evaluate the efficacy of prophylactic sartorius flaps, compared to layered closure, in preventing 30-day hospital readmission for deep space infection. Secondarily, this study evaluated the effect of oblique versus vertical groin incisions on 30-day hospital readmission for deep space infection.

## Materials and methods

This was a single-center retrospective study of all patients of a single board-certified vascular surgeon who underwent open lower extremity revascularization procedures involving groin incisions for femoral artery exposure from 2017-2021. Patients with active groin infections were excluded. Patients who received prophylactic sartorius flaps were compared to those with layered closure alone in terms of the primary outcome of 30-day hospital readmission for DSI. Deep space infection was defined per the Samson Classification as group three or greater [2] (Table 1).

**Table 1:** Classification for peripheral arterial prosthetic grafts as described by Samson et al. [2].

Samson Classification	
Group	Description
1	Superficial to dermis
2	Subcutaneous tissues, no graft contact
3	Direct graft contact, no anastomosis involvement
4	Direct anastomosis contact, no bacteremia or anastomotic bleeding
5	Surrounds anastomosis with sepsis or anastomotic bleeding

**Figure 1:**
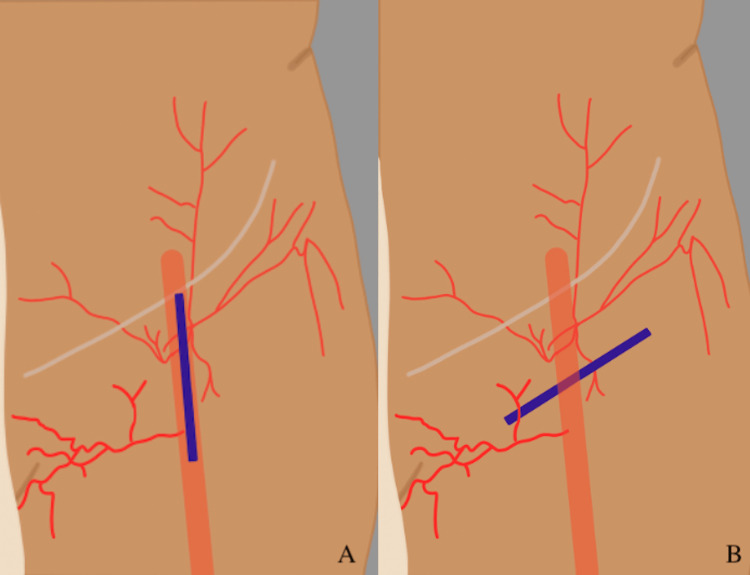
Position of incision for femoral artery exposure. A) Vertical incision B) Oblique incision

**Figure 2:**
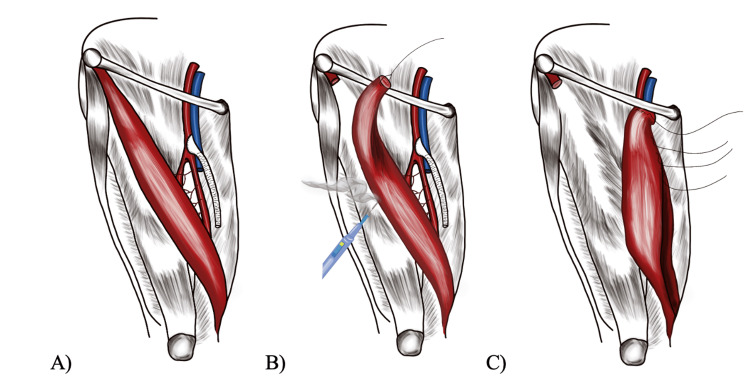
Surgical steps of sartorius flap. A) Native position of sartorius muscle in proximity to femoral to distal artery bypass graft B) Lateral mobilization of the sartorius muscle C) Medial rotation of sartorius muscle flap to ensure coverage of femoral vessels and bypass anastomosis

Secondarily, patients with oblique versus vertical incision orientation (Figure 1) were compared irrespective of the presence of sartorius flaps to determine the effect on the primary outcome. Sartorius flaps involved the separation of the muscle from the anterior superior iliac spine, medial rotation of the muscle over the common femoral artery, and medial anchoring with 2-0 Vicryl figure-of-eight sutures. These surgical steps ensured coverage of any underlying patch angioplasty or peripheral bypass (Figure 2).

The subsequent soft tissue closure steps were identical to the layered closure steps. Layered closure involved three layers, including topical hemostatic agents followed by a running 2-0 Vicryl for the femoral sheath, a 3-0 Vicryl interrupted deep subcutaneous layer, a 4-0 Monocryl subcuticular layer, and skin covered with Dermabond. The use of incisional negative pressure wound vacuum therapy (PREVENA™, KCI, US) for seven days postoperatively was determined at the discretion of the attending surgeon; however, was used nearly unanimously across all groups.

The decision for or against sartorius flaps was determined by the attending surgeon if a patient was in the Penn Groin Assessment Scale intermediate or high-risk categories or was in the low-risk category with additional risk factors of female gender and diabetes mellitus. There was no mandatory postoperative bed rest period, and patients were encouraged to ambulate postoperative day one. Patient demographics, comorbidities, and postoperative complications were compared between the muscle flap and layered closure groups and also oblique versus vertical incision groups.

The experimental protocol was approved by the Institutional Review Board and was exempt from full board review based on its retrospective nature. Study data were imported into IBM Corp. Released 2017. IBM SPSS Statistics for Windows, Version 25.0. Armonk, NY: IBM Corp. and summarized separately by the presence of muscle flap and incision orientation. Means and standard deviations summarized numeric data, while frequencies and percentages summarized categorical data by stratum. Numeric outcome data were compared for mean equality between strata via independent samples t-tests. Categorical data employed either Fisher’s exact or Pearson chi-square tests to compare strata for distributional equality. The categorical outcome for readmission timing was compared for equivalence using exact Kendall’s tau tests to account for the ordinal scale. All statistical testing was two-sided, with p<0.05 considered statistically significant.

## Results

One hundred seventy-five patients were included in this study. Fifty-three patients received sartorius flaps, and 122 received layered closure only (Table 2). Seventy patients had oblique incisions, and 105 had vertical incisions (Table 3).

When comparing the demographics of the sartorius flap group to the layered closure group, we found there to be a higher rate of previous groin surgery in the flap group (45.3% vs. 24.7%, p<0.01). The remaining demographics were similar in each group, including mean BMI, sex, smoking history, diabetes, chronic kidney disease (CKD), obesity, and age (Table 2).

**Table 2:** Patient demographics in layered closure versus sartorius flap groups. CAD: Coronary artery disease

Patient Demographics			
	Type of Closure		
	Layered Closure (n=122)	Sartorius Flap (n=53)	P-value
Mean Age (SD)	64.2 (12.43)	65.4 (10.59)	0.537
Female (%)	44 (36.1)	25 (47.2)	0.167
Male, n (%)	78 (63.9)	28 (52.8)	
Mean BMI (SD)	27.3 (8.68)	26.0 (8.19)	0.346
Smoking History, n (%)	74 (61.2)	27 (50.9)	0.209
Diabetes (%)	36 (29.5)	16 (30.2)	0.928
Heart Failure, n (%)	33 (27.0)	11 (20.8)	0.378
Hypertension, n (%)	84 (68.9)	38 (71.7)	0.707
CAD, n (%)	68 (55.7)	27 (50.9)	0.559
Cerebrovascular Disease, n (%)	24 (19.7)	13 (24.5)	0.47
Chronic Kidney Disease, n (%)	20 (16.4)	11 (20.8)	0.487
Obesity, n (%)	18 (14.8)	8 (15.1)	0.954
Prior Groin Surgery, n (%)	30 (24.6)	24 (45.3)	0.006
Trauma Patient, n (%)	5 (4.1)	2 (3.8)	1

The vertical incision group had a higher rate of previous groin surgery (38.1% vs. 20.0%, p<0.02), while the oblique incision group had a higher rate of obesity as defined by BMI>30 (24.3% vs. 8.6%, p<0.01). The remaining patient demographics were similar in each group, including mean BMI, sex, smoking history, diabetes, CKD, and age (Table 3).

**Table 3:** Patient demographics in oblique versus vertical incision groups.

Demographics			
	Incision Orientation		
	Oblique (N=70)	Vertical (N=105)	P-value
Mean Age (SD)	63.9 (14.63)	64.9 (9.70)	0.605
Female, n (%)	27 (39.6)	42 (40.0)	0.85
Male, n (%)	43 (61.4)	63 (60.0)	
Mean BMI (SD)	28.2 (9.65)	26.0 (7.62)	0.101
Smoking History, n (%)	41 (58.6)	60 (57.7)	0.908
Diabetes n (%)	19 (27.1)	33 (31.4)	0.543
Heart Failure, n (%)	15 (21.4)	29 (27.6)	0.355
Hypertension, n (%)	51 (72.9)	71 (67.6)	0.46
CAD, n (%)	38 (54.3)	57 (54.3)	1
Cerebrovascular Disease, n (%)	14 (20.0)	23 (21.9)	0.762
Chronic Kidney Disease, n (%)	9 (12.9)	22 (21.0)	0.169
Obesity, n (%)	17 (24.3)	9 (8.6)	0.004
Prior Groin Surgery, n (%)	14 (20.0)	40 (38.1)	0.011
Trauma Patient, n (%)	4 (5.7)	3 (2.9)	0.44

Regarding the primary outcome, there were nine total 30-day readmissions for DSI across all groups. There was a lower rate of DSI in the sartorius flap group (1.9% vs. 6.6%, p=0.280), although this was not statistically significant. There was no difference in one-year mortality between the two groups (Table 4).

**Table 4:** Outcomes in layered closure versus sartorius flap groups.

Outcomes			
	Layered Closure (n=122)	Sartorius Flap (n=53)	P-value
Deep Space Infection, n (%)	8 (6.6)	1 (1.9)	0,280
1-Year Mortality, n (%)	17 (13.9)	9 (17.0)	0.603

There was no difference in 30-day readmission for DSI between the oblique and vertical incision groups at 4.3% and 5.7% (p=0.743), respectively. Further, there was no difference in one-year mortality at 15.7% and 14.3%, respectively (p=0.795) (Table 5).

**Table 5:** Outcomes in oblique versus vertical incision groups.

	Outcomes		
	Oblique (n=70)	Vertical (n=105)	P-value
Deep Space Infection, n (%)	3 (4.3)	6 (5.7)	0.743
1-Year Mortality, n (%)	11 (15.7)	15 (14.3)	0.795

## Discussion

Prophylactic local muscle flaps for preventing groin wound complications have gained popularity in recent years, particularly in the high-risk vascular surgery patient population. Prior studies have demonstrated efficacy in preventing complications. Fischer et al. demonstrated patients undergoing peripheral revascularization surgery had a significantly lower rate of both overall wound complication and infection if prophylactic sartorius flaps were conducted; however, the authors defined deep infection as simply involving muscle or fascia [7]. In our experience, an important delineation for deep infections is the involvement of the femoral vessels or bypass, thus jeopardizing the recent operation. Similar to our study, Price et al. included the high-risk population of reoperative groins; however, this study included both sartorius and gracilis flaps. The authors abandoned the sartorius flap in the latter half of the study in favor of the gracilis flap after noting a 32% complication rate with the sartorius flaps. They rationalized that significant superficial femoral artery disease compromises the vascular pedicle to the sartorius and places the flap at risk for ischemia, whereas the gracilis vascular pedicle arises from the profunda femoris artery [4]. Wallace et al. not only required an unnecessary five-day postoperative bed rest period but also excluded any reoperative groin to evaluate a truly “prophylactic” muscle flap [8]. In our experience, the lack of reoperative groins excludes one of the most significant risk factors for postoperative groin complications.

One of the few studies to evaluate groin complications in regard to oblique versus vertical incisions is Maeda et al. [9]. The authors demonstrated a decreased complication rate in the oblique group. However, incisions were for endovascular delivery of aortic stent grafts rather than femoral exposure for endarterectomy or peripheral bypass.

We present a five-year retrospective study comparing prophylactic sartorius flaps versus layered closure alone and oblique versus vertical incision orientation. We aimed to look at a broad representation of the vascular surgery population utilizing sartorius flaps only. Sartorius flaps are preferred at our institution to gracilis flaps given the ease of exposure, minimal increase in overall operative time, and minimal postoperative patient functional deficits. The incision for a sartorius flap needs minimal additional cephalad extension for separation of the origin on the anterior superior iliac spine (ASIS), compared to the additional lateral incision required for the harvest of a gracilis flap. We further aimed to focus solely on DSI with a strict definition of directly involving the femoral vessels or adjacent bypass. This definition of deep space infection is important as it places the limb at risk with a potential need for morbid interventions such as bypass revision, excision of the prosthesis, or limb amputation.

We noted a significantly increased rate of reoperative groins in the sartorius flap compared to layered closure groups (45% vs. 25%, p<0.01). When comparing the primary outcome of 30-day readmission for DSI, the rate in the sartorius flap group was decreased compared to the layered closure (1.9% vs. 6.6%), although this was not statistically significant (p=0.280). It should be noted that the single patient with a DSI following sartorius flap was cachectic from prior chemotherapy and had tissue cultures positive for Serratia; therefore, this case was likely complicated by immunosuppression and a highly virulent organism. Nonetheless, at minimum, we noted the sartorius flaps were performed in a higher-risk population, as evidenced by an increased rate of reoperative groins, and still experienced DSIs at a similar rate to the lower risk-layered closure group. Furthermore, it is reasonable to deduce that protection with vascularized sartorius flaps prevents bacteria in more superficial SSIs from reaching the femoral vessels or underlying bypasses, thus avoiding the need for additional revascularization operations.

Regarding incision orientation, the oblique incision patient population had a higher rate of obesity (24.3% vs. 8.6%, p<0.01). However, the vertical incision group had a higher percentage of reoperative groins compared to oblique incisions (38.1 vs. 20.0%, p=0.01). As there was no difference in 30-day readmission for DSI in these groups and there were conflicting distributions of high-risk factors, we feel incision orientation did not have a significant effect on the primary outcome in our patient population.

There were limitations to this study. The study was underpowered as a total of 731 patients are required to attain 80% power. The small sample size to detect a difference in a relatively rare outcome resulted in an underpowering of the statistical testing, thus prone to a type II statistical error. Additionally, this was a retrospective, single-surgeon study with an uncontrolled selection of which patients received muscle flaps. A larger, randomized controlled trial with multiple surgeons would be ideal in future studies.

## Conclusions

Patients undergoing lower extremity revascularization procedures involving groin incisions are at high risk for wound complications, with deep space infection being the most concerning for potential limb loss. Our institution has experienced promising results in preventing DSI with prophylactic sartorius flaps, especially in patients with high-risk characteristics. We found an overall decreased rate of DSI in patients with sartorius flaps, although further evaluation with increased sample size is required for adequate study power. We believe sartorius flaps are a relatively simple solution for preventing the morbidity associated with postoperative groin complications.
